# Patulin Stimulates Progenitor Leydig Cell Proliferation but Delays Its Differentiation in Male Rats during Prepuberty

**DOI:** 10.3390/toxins15090581

**Published:** 2023-09-20

**Authors:** Huitao Li, Ming Su, Hang Lin, Jingjing Li, Shaowei Wang, Lei Ye, Xingwang Li, Renshan Ge

**Affiliations:** 1 Department of Anesthesiology and Perioperative Medicine, The Second Affiliated Hospital and Yuying Children’s Hospital, Wenzhou Medical University, Wenzhou 325027, China; lihuitao1991@163.com (H.L.); suming2000@163.com (M.S.); lh1014794144@163.com (H.L.); lbling68@163.com (J.L.); smilewei9898@163.com (S.W.); leiye3567@163.com (L.Y.); li-xingwang@163.com (X.L.); 2 Key Laboratory of Pediatric Anesthesiology, Ministry of Education, Wenzhou Medical University, Wenzhou 325027, China; 3 Key Laboratory of Environment and Male Reproductive Medicine of Wenzhou, Key Laboratory of Structural Malformations in Children of Zhejiang Province, The Second Affiliated Hospital and Yuying Children’s Hospital, Wenzhou Medical University, Wenzhou 325027, China

**Keywords:** mycotoxin, testosterone, progesterone, cell cycle, steroidogenesis

## Abstract

Patulin is a mycotoxin with potential reproductive toxicity. We explored the impact of patulin on Leydig cell (LC) development in male rats. Male Sprague Dawley rats (21 days postpartum) were gavaged patulin at doses of 0.5, 1, and 2 mg/kg/day for 7 days. Patulin markedly lowered serum testosterone at ≥0.5 mg/kg and progesterone at 1 and 2 mg/kg, while increasing LH levels at 2 mg/kg. Patulin increased the CYP11A1^+^ (cholesterol side-chain cleavage, a progenitor LC biomarker) cell number and their proliferation at 1 and 2 mg/kg. Additionally, patulin downregulated *Lhcgr* (luteinizing hormone receptor), *Scarb1* (high-density lipoprotein receptor), and *Cyp17a1* (17α-hydroxylase/17,20-lyase) at 1 and 2 mg/kg. It increased the activation of pAKT1 (protein kinase B), pERK1/2 (extracellular signal-related kinases 1 and 2), pCREB (cyclic AMP response binding protein), and CCND1 (cyclin D1), associated with cell cycle regulation, in vivo. Patulin increased EdU incorporation into R2C LC and stimulated cell cycle progression in vitro. Furthermore, patulin showed a direct inhibitory effect on 11β-HSD2 (11β-hydroxysteroid dehydrogenase 2) activity, which eliminates the adverse effects of glucocorticoids. This study provides insights into the potential mechanisms via which patulin affects progenitor LC development in young male rats.

## 1. Introduction

Patulin is a mycotoxin produced by certain fungi, such as *Penicillium* and *Aspergillus* [1]. It is commonly found in moldy fruits, especially apples and apple products, and can cause food spoilage [1]. Exposure to patulin can occur through the consumption of contaminated food, the inhalation of contaminated dust or aerosols during food processing, and skin contact [2]. The average intake of patulin varies depending on the type and quantity of consumed fruits and fruit products. Its levels are often highest in apple products, especially apple juice and cider [3]. The World Health Organization (WHO) has set the maximum permissible level of patulin in apple juice at 50 μg/kg [4]. Patulin can exhibit acute and chronic toxic effects. Acute toxicity includes gastrointestinal disturbances such as nausea, vomiting, and diarrhea. In severe cases, it may cause liver damage [5]. Patulin has been extensively studied for its toxic effects on human health, including its potential impact on Leydig cell (LC) development [1,6,7].

LCs mainly secrete testosterone, an androgen [8]. LC development starts during prepuberty, and progenitor LCs emerge at day 21 postpartum (PND21) [8]. The progenitor LCs then proliferate and undergo maturation into immature LCs, which eventually reach approximately half the mature LC number [8]. The proliferation of interstitial progenitor cells in the prepubertal stage is crucial for the development of LCs in the testes. One vital component involved in this process is CCND1, a cyclin that acts as a receptor for external signals triggering cell proliferation [9]. It shows high expression levels in progenitor LCs [10]. The activity of CCND1 is regulated by inhibitory pathways, including the CDKN1B (P27) protein, which functions as an inhibitor of the CCND1–CDK complex [11,12]. It is worth noting that the knockout of CCND1 has been linked to LC proliferation [13]. TP53, a master transcription factor, plays a role in suppressing cell proliferation by upregulating CDKN1B levels [14]. Moreover, factors such as pAKT1, pERK1/2, and pCREB have been implicated in the activation of cell proliferation [15,16,17].

Androgen synthesis, which involves the conversion of cholesterol to testosterone, begins during this maturation process and involves steroidogenic machinery cascades, including luteinizing hormone/choriogonadotropin receptor (LHCGR) signaling, cholesterol uptake via scavenger receptor class B member 1 (SCARB1), cholesterol shuttle between the cytoplasm and mitochondria via steroidogenic acute regulatory protein (STAR), cytochrome P450 family 11 member A1 (CYP11A1), 3β-hydroxysteroid dehydrogenase 1/Δ^5,4^-isomerase (HSD3B1), cytochrome P450 family 17 member A1 (CYP17A1), as well as 17β-hydroxysteroid dehydrogenase isoform 3 (HSD17B3) [8]. Immature LCs also abundantly express steroid 5α-reductase 1 (SRD5A1) and aldo-keto reductase C14 (AKR1C14) [18]. Furthermore, 11β-hydroxysteroid dehydrogenase type 2 (11β-HSD2) is also closely associated with the development of LCs. Previous studies indicate that with the maturation of LCs, 11β-HSD2 expression is significantly upregulated, and it catalyzes the inactivation of active glucocorticoid corticosterone [19,20], which inhibits the development of LCs via glucocorticoid receptor signaling [21].

Multiple studies have indicated a correlation between the recent increase in male infertility and the rising levels of mycotoxins in the environment [22]. Patulin, as one of these mycotoxins, has been demonstrated to have toxic effects on LC development [23]. Patulin exposure to pubertal male rats for 60 or 90 days causes an increase in testosterone levels and LH levels for 90 days, LC hyperplasia in the interstitial tissue, and the disorganization of the seminiferous tubule [24].

The prepubertal period involves the rapid onset of the multiplication of progenitor LCs and the transition from progenitor LCs into immature LCs. However, whether patulin affects LC development in prepuberty is still unclear. The objective of this study was to address patulin’s toxic effects on LC development and the potential mechanisms.

## 2. Results

### 2.1. The Overt Toxicity of Patulin

After 7 days of treatment, patulin had no effects on body weight and testis weight (Table 1). This compound did not affect the behaviors of animals.

### 2.2. Patulin Reduces Testosterone and Progesterone Levels In Vivo

**Figure 1:**
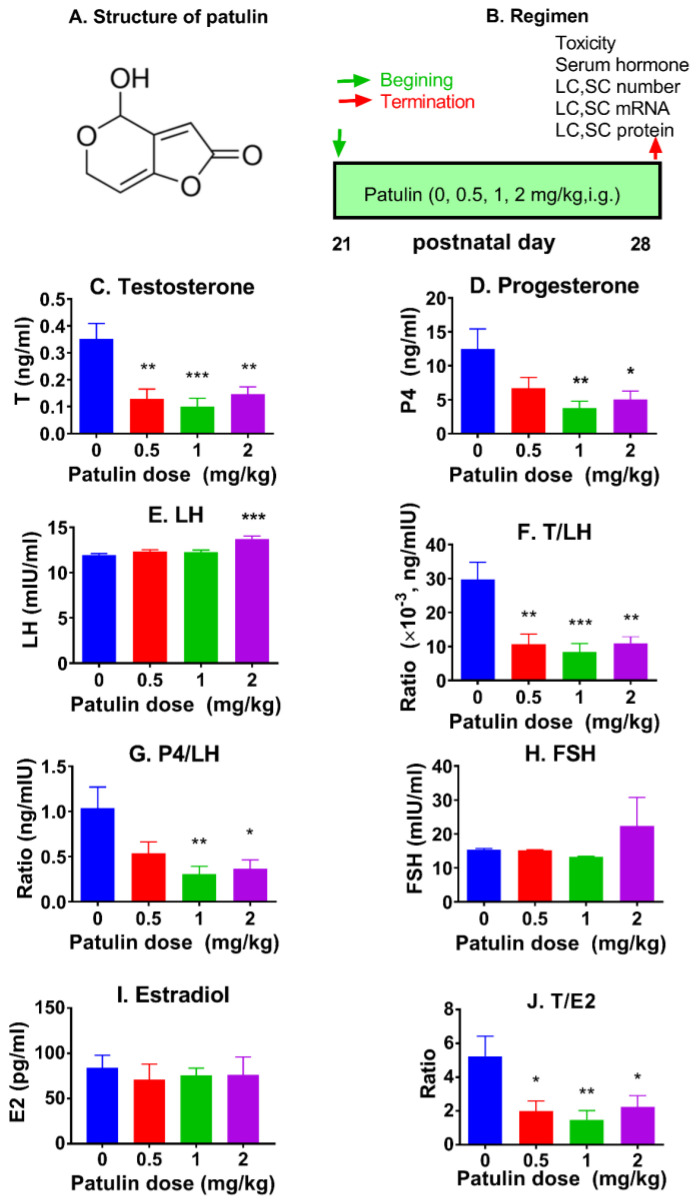
Structure of patulin, regimen, and hormonal levels. Chemical structure of patulin (**A**); regimen of patulin (**B**) (in vivo, including Figure 1, Figure 2, Figure 3, Figure 4, Figure 5 and Figure 6); and serum testosterone (T) (**C**), progesterone (P4) (**D**), LH (**E**), T/LH ratio (**F**), P4/LH ratio (**G**), FSH (**H**), estradiol (E2) (**I**), and T/E2 ratio (**J**) levels. Means ± SEM; n = 6. * *p* < 0.05, ** *p* < 0.01, and *** *p* < 0.001 show significant differences from the vehicle control.

We analyzed serum hormone levels. The results reveal that patulin reduced serum testosterone levels at ≥0.5 mg/kg (*p* < 0.01 or 0.001; Figure 1C) and progesterone levels at 1 and 2 mg/kg (*p* < 0.01 or 0.05; Figure 1D). Patulin markedly increased the LH amount at 2 mg/kg (Figure 1E). We further estimated the testosterone/LH and progesterone/LH ratios and found that patulin caused a significant reduction in the testosterone/LH ratio at 0.5 mg/kg and higher and the progesterone/LH ratio at 1 and 2 mg/kg (Figure 1F,G). However, patulin did not affect serum FSH levels, although its level was slightly elevated at 2 mg/kg (Figure 1H). We measured the serum estradiol (E2) output and estimated the testosterone-to-estradiol (T/E2) ratio (Figure 1I,J) and found that patulin had no impact on serum estradiol levels but markedly lowered the T/E2 ratio at ≥0.5 mg/kg. These results indicate that patulin directly disrupts LC development in the testis.

### 2.3. Patulin Increases Progenitor LC Number but Not the More Mature LC Number

**Figure 2:**
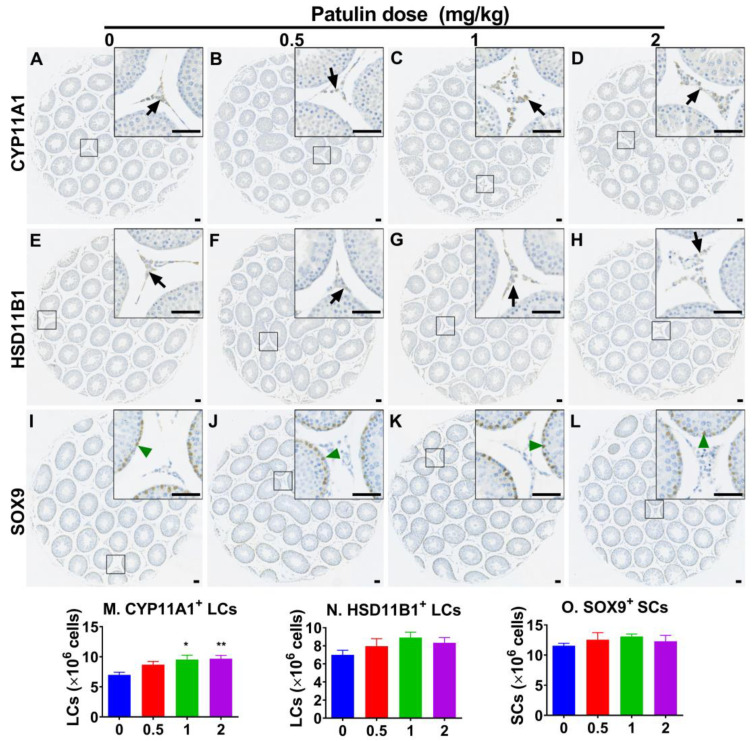
The effect of LC and Sertoli cell numbers after in vivo patulin exposure. (**A**–**D**): CYP11A1^+^ LCs; (**E**–**H**): HSD11B1^+^ LCs; (**I**–**L**): SOX9^+^ Sertoli cells. (**A**,**E**,**I**): control; (**B**,**F**,**J**): 0.5 mg/kg/day patulin; (**C**,**G**,**K**): 1 mg/kg/day patulin; (**D**,**H**,**L**): 2 mg/kg/day patulin; (**M**,**N**,**O**): quantitative results. Means ± SEM; n = 6 (randomly selected). * *p* < 0.05 and ** *p* < 0.01 show significant differences from the vehicle control. Black arrows designate LCs. Green arrowheads designate Sertoli cells. Scale bar = 50 μm.

We used CYP11A1 as an LC marker in this lineage and found that patulin increased the CYP11A1^+^ LC number at 1 and 2 mg/kg (*p* < 0.05 and 0.01; Figure 2). We used HSD11B1 as a more mature LC marker and witnessed that patulin exhibited no effect on the HSD11B1^+^ LC number (Figure 2). These results indicate that the increased CYP11A1^+^ cells were progenitor LCs. We also found that patulin had no effect on the SOX9-labeled Sertoli cell number after using the Sertoli cell marker SOX9 (Figure 2).

### 2.4. Patulin Increases Progenitor LC Proliferation

**Figure 3:**
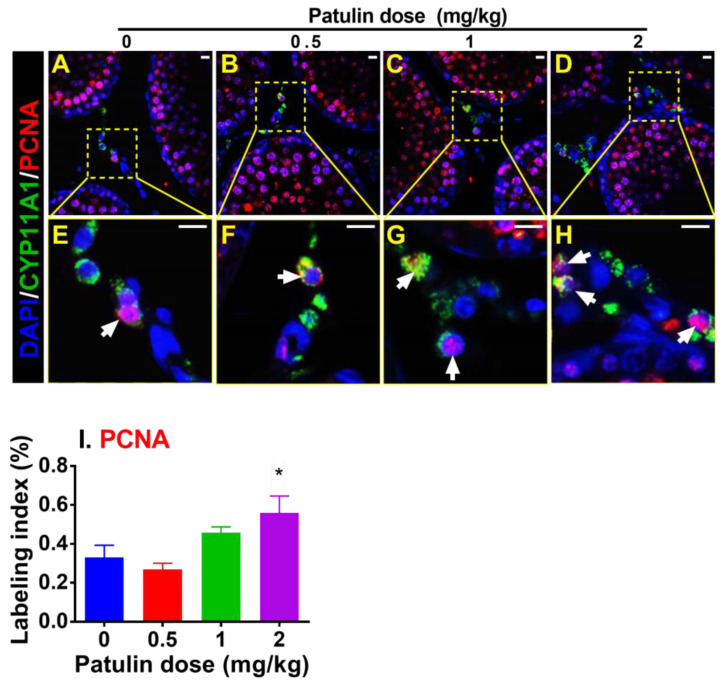
Immunofluorescence staining of LC proliferation biomarker from rat testis after in vivo patulin exposure. (**A**–**D**): Immunofluorescence images of sections from rat testis after exposure to different doses of patulin (0, 0.5, 1, and 2 mg/kg/day). Each panel represents a different patulin concentration. (**E**–**H**): Enlarged images of specific regions within (**A**–**D**), indicated by yellow squares, highlighting the staining of PCNA (red—nucleus, proliferation biomarker) and CYP11A1 (green—cytoplasm, LC biomarker) and DAPI (blue—nucleus, counterstain). (**I**): Quantitative data for PCNA labeling (white arrow) of LCs. Means ± SEM; n = 6. * *p* < 0.05 shows a significant difference from the vehicle control. Scale bar = 20 μm.

Since patulin induces LC hyperplasia, PCNA was adopted as a proliferating cell marker and CYP11A1 as an LC marker for the LC labeling index. Patulin enhanced LC proliferation at 1 and 2 mg/kg (*p* < 0.05 and 0.01; Figure 3). These results indicate that patulin increases the proliferation of progenitor LCs.

### 2.5. Patulin Alters LC Gene Expression

**Figure 4:**
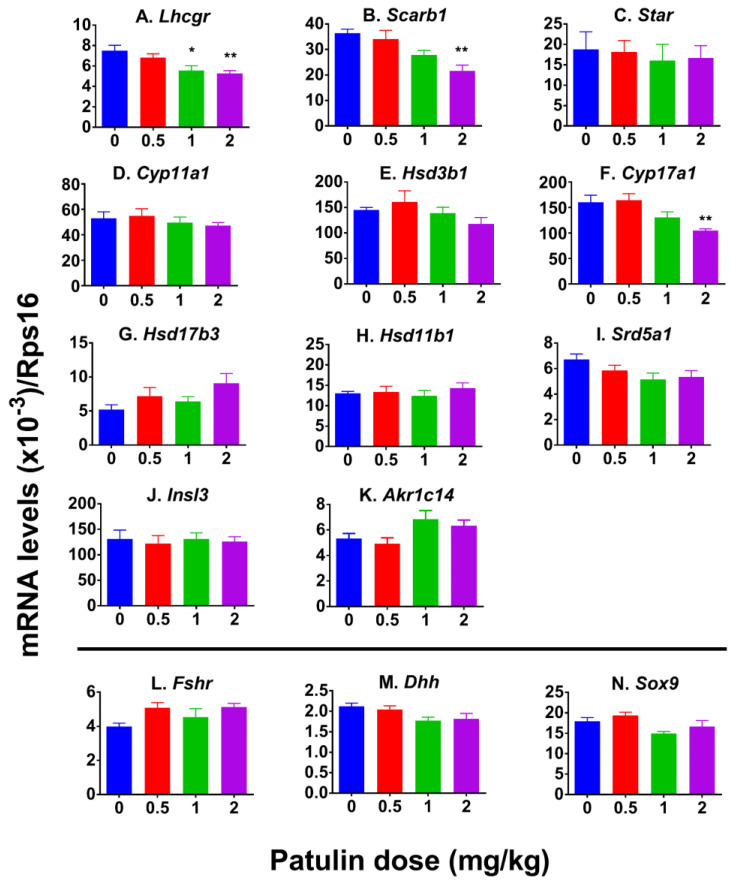
Effects of patulin on mRNA expression in rat testis after in vivo patulin exposure. The testicular mRNA levels were measured using qPCR and adjusted to CYP11A1^+^ LC number for LC genes. (**A**–**K**): LC genes (*Lhcgr*, *Scarb1*, *Star*, *Cyp11a1*, *Hsd3b1*, *Cyp17a1*, *Hsd17b3*, *Hsd11b1*, *Srd5a1*, *Insl3*, and *Akr1c14*); (**L**–**N**): Sertoli cell genes (*Fshr*, *Dhh*, and *Sox9*). Means ± SEM; n = 6. * *p* < 0.05 and ** *p* < 0.01 show significant differences from vehicle control.

In rat testis, *Lhcgr*, *Star*, *Scarb1*, *Hsd3b1*, *Cyp11a1*, *Cyp17a1*, *Hsd17b3*, *Srd5a1*, *Akr1c14*, *Insl3*, and *Hsd11b1* are exclusively located in LCs, hereby referred to as LC genes, and *Sox9*, *Fshr*, and *Dhh* are exclusively expressed in Sertoli cells, hereby referred to as Sertoli cell genes. Patulin markedly upregulated *Hsd17b*, and *Hsd11b1* mRNA expression at 2 mg/kg and elevated *Akr1c14* at 1 and 2 mg/kg (Figure S1), suggesting that patulin alters LC gene expression. However, patulin did not affect Sertoli cell gene expression. Since patulin increased the LC number, the LC number was selected as the adjustment for LC mRNA per se. The results show that patulin downregulated *Lhcgr*, *Scarb1*, and *Cyp17a1* per LC (Figure 4), indicating that patulin delays LC differentiation. Patulin did not change *Sod1*, *Sod2*, *Gpx1*, and *Cat* mRNA expression and MDA levels (Figure S2), indicating that the patulin-mediated suppression of LC development is not via oxidative stress induction.

### 2.6. Patulin Alters LC Protein Expression

**Figure 5:**
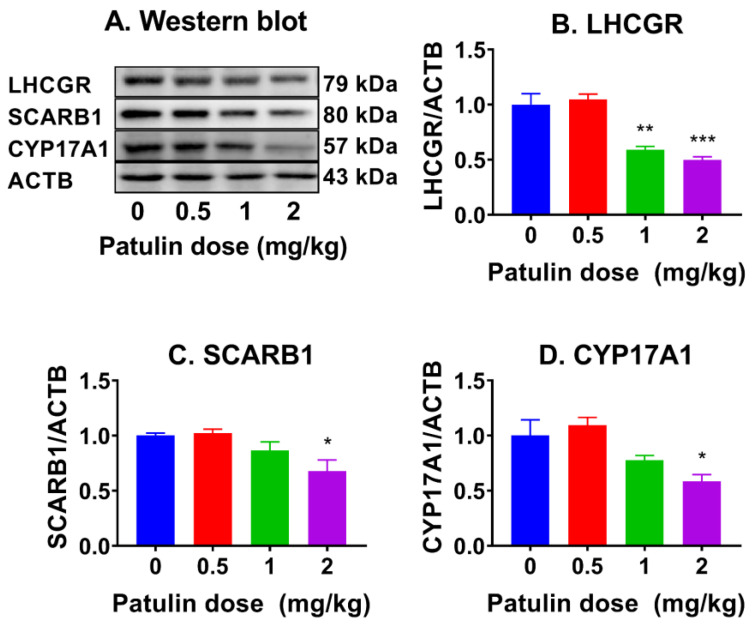
Levels of LC proteins in the testis after in vivo patulin exposure. (**A**): Western blot image; (**B**–**D**): Quantification of protein levels of LHCGR, SCARB1, CYP17A1 after normalized to ACTB (internal control). Mean ± SEM, n = 3–7 (randomly selected samples). * *p <* 0.05, ** *p <* 0.01, and *** *p <* 0.001 show significant differences from vehicle control.

LHCGR, SCARB1, and CYP17A1 were estimated using Western blotting, and the results show that patulin significantly reduced LHCGR, SCARB1, and CYP17A1 at 1–2 mg/kg (Figure 5), conforming to their mRNA changes.

### 2.7. Patulin Increases Proliferation-Related Protein and Signaling Protein Expression

**Figure 6:**
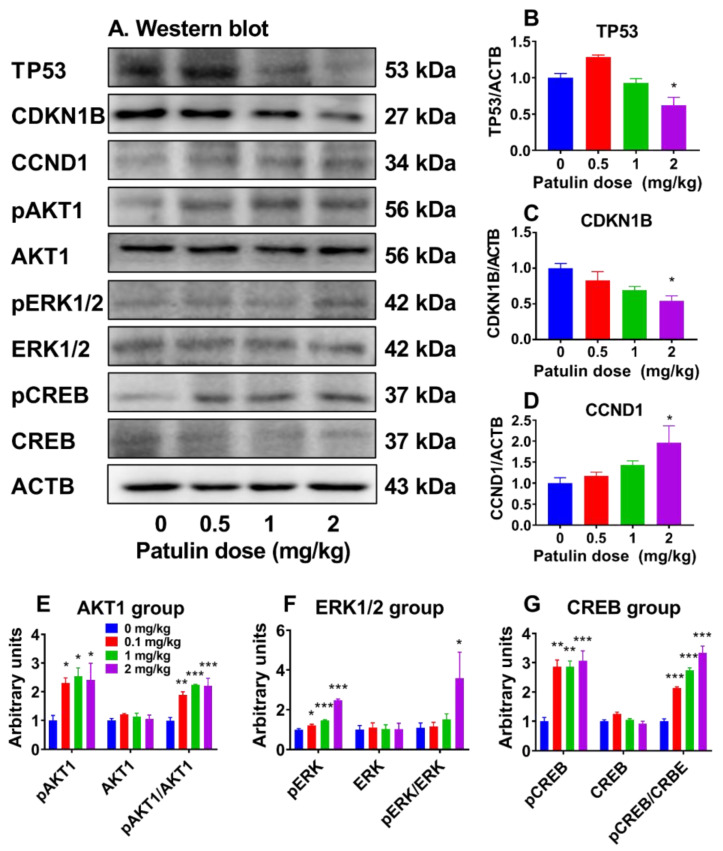
Levels of signal proteins in the testis after in vivo patulin exposure. (**A**): Western blot image; (**B**–**G**): quantitative results. Protein levels were adjusted to ACTB (internal control). Means ± SEM; n = 4–9 (randomly selected testis samples). * *p* < 0.05, ** *p* < 0.01, and *** *p* < 0.001 show significant differences from vehicle control.

We performed Western blotting to gauge the protein levels of proliferation-related proteins and found that patulin significantly increased cyclin D1 (CCND1) at 2 mg/kg while decreasing proliferation-inhibitory protein TP53 and CDKN1B at 2 mg/kg, indicating that patulin increases LC proliferation via downregulating the proliferation inhibition pathway of TP53–DKN1B–CCND1 signaling. To further investigate the proliferation-related signaling pathways, we measured the phosphorylated and total levels of AKT1, ERK1/2, and CREB. It was found that patulin elevated the phosphorylation of AKT1, ERK1/2, and CREB (Figure 6), indicating that patulin also stimulates cell proliferation via proliferation stimulation pathways.

### 2.8. Effects of Patulin on Androgen Secretion and Proliferation of Rat R2C Cells

**Figure 7:**
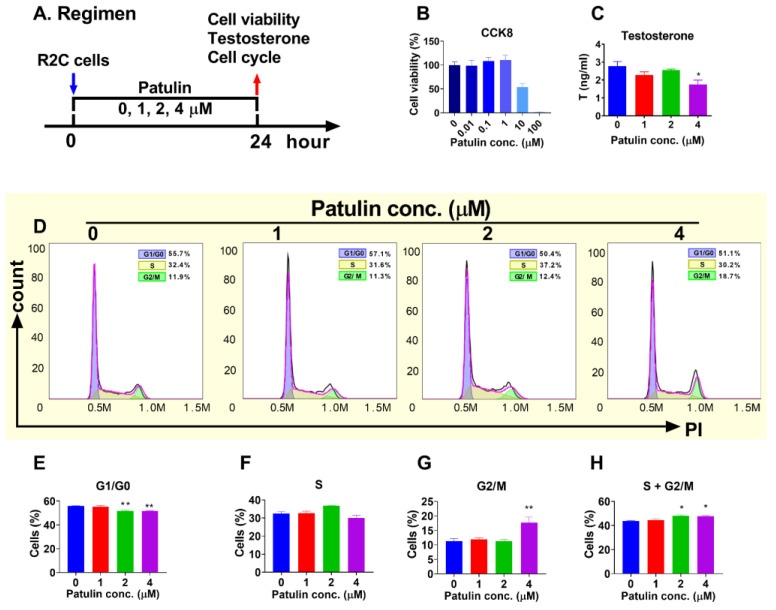
Patulin in vitro treatment regimen and cell viability, testosterone levels, and cell cycle analysis of R2C cells after patulin treatment for 24 h. (**A**): regimen; (**B**): cell viability (CCK8); (**C**): medium testosterone; (**D**): flow cytometry analysis of cell cycle; (**E**–**H**): quantification for G0/G1, S, G2/M, and S + G2/M phase populations, respectively. Means ± SEM; n = 3. * *p* < 0.05 and ** *p* < 0.01 show significant differences from the control.

**Figure 8:**
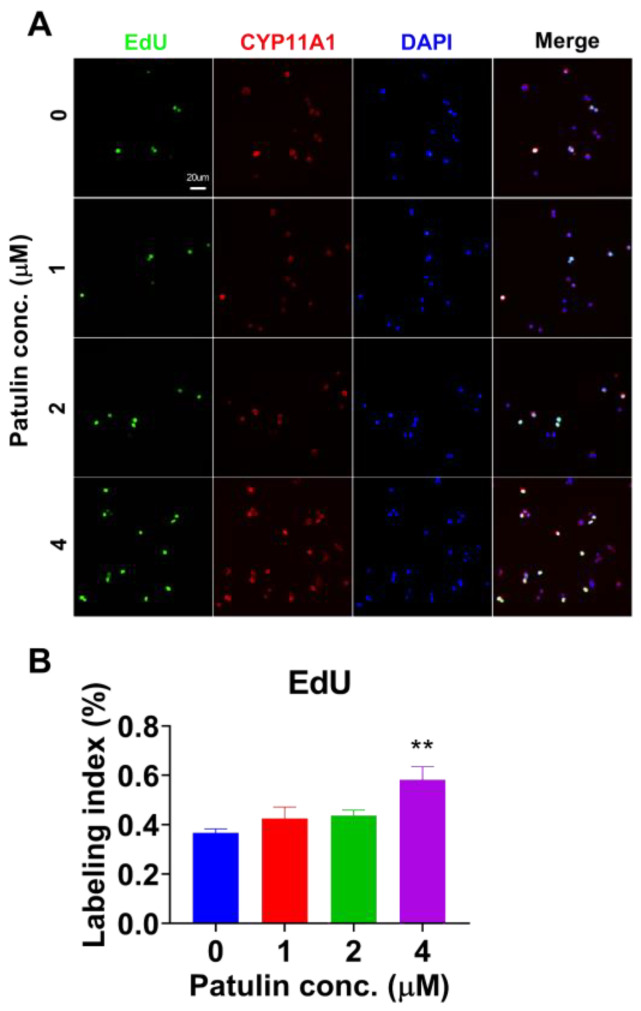
EdU incorporation into proliferative R2C cells after patulin in vitro treatment. (**A**): EdU (green nucleus), CYP11A1 (red cytoplasm), and DAPI (blue counterstain) in R2C cells after patulin treatment; (**B**): quantification of EdU labeling in R2C cells. Means ± SEM; n = 3. ** *p* < 0.01 shows a significant difference from the control.

We tested which concentration range of patulin has no cytotoxic action after 24 h of treatment (Figure 7A). The results revealed that patulin had no significant effect on cell viability up to 10 μM, as assessed using the CCK8 assay (Figure 7B). Then, we used a range of concentrations of patulin, at which patulin did not cause cytotoxicity. Indeed, at a concentration of 4 μM, patulin significantly reduced testosterone secretion (Figure 7C). We further analyzed whether patulin affected cell proliferation. The results demonstrate that patulin significantly stimulated G2/M progression (Figure 7D–H), indicating an influence on the cell cycle. This effect was further supported by the increase in cell proliferation observed using the EdU incorporation assay (Figure 8), which measures DNA synthesis during cell division.

### 2.9. Patulin Directly Inhibits 11β-HSD2 Activity

**Figure 9:**
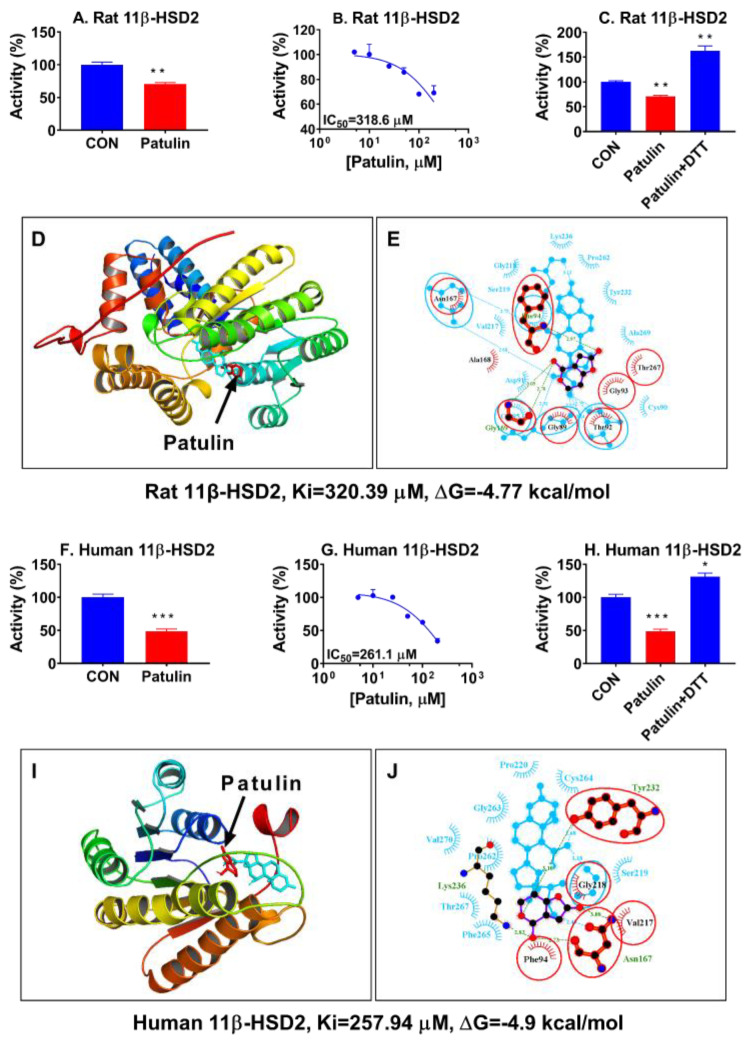
Patulin’s inhibition, IC50, dithiothreitol (DTT) effect, and docking analysis of rat and human 11β-HSD2 enzymes. (**A**,**F**): Inhibition by patulin of rat and human 11β-HSD2, respectively; (**B**,**G**): IC_50_ values for patulin for rat 11β-HSD2 and human 11β-HSD2, respectively; (**C**,**H**): effects of DTT on patulin-induced inhibition of rat and human 11β-HSD2, respectively. (**D**,**I**): Superimposed images of rat or human 11β-HSD2 with patulin and substrate cortisol, respectively. Red = patulin; blue = substrate cortisol. (**E**,**J**): Superimposed images of rat or human 11β-HSD2 with patulin and substrate cortisol, respectively. Purple = patulin, cyan = substrate cortisol, red circle = common contacting residues, and green dash line = hydrogen bonds. The Ki and ΔG values obtained through docking analysis are displayed beneath the panels of the figure, respectively, which contain the 3D/2D superimposed images. Means ± SEM; n = 4. * *p* < 0.05, ** *p* < 0.01, and *** *p* < 0.001 show significant differences from the control.

We performed enzyme inhibition, and it was found that patulin significantly inhibited 11β-HSD2 activity at 100 μM (Figure 9A). Further analysis showed that the inhibitory strength (IC_50_) of patulin was 318.2 μM (Figure 9B). To understand the mechanism, we used the reducing agent DTT to protect the SH group of 11β-HSD2. Indeed, patulin can completely reverse the inhibition of 11β-HSD2 (Figure 9C), indicating patulin inhibits 11β-HSD2 activity via attacking the SH group of catalytic residues. Docking analysis revealed that patulin potentially contacts the steroid active site by attacking the SH groups (Figure 9D,E). Additionally, the obtained inhibition constant (Ki) of patulin for the 11β-HSD2 enzyme (320.39 μM) was similar to the previously mentioned IC50 values. To determine if patulin exhibited similar inhibition of human 11β-HSD2 from testis samples, it was found that, indeed, patulin inhibited the activity of human 11β-HSD2 at a concentration of 100 μM (Figure 9F). Furthermore, dose–response analysis revealed that the IC50 value of patulin was 261.1 μM, which was close to the Ki value of 257.94 μM obtained from the docking analysis (Figure 9G). The addition of DTT can reverse the inhibition by patulin, indicating that patulin inhibits human 11β-HSD2 via targeting the SH group (Figure 9H). The docking analysis for patulin with human 11β-HSD2 revealed that patulin interacts with the Cys90 of human 11β-HSD2 (Figure 9I,J), indicating that patulin possibly interacts with the critical residue Cys90.

## 3. Discussion

The findings of this study reveal that patulin exposure resulted in significant alterations in hormone levels with reductions in serum testosterone and progesterone levels and elevation in LH levels. Although patulin stimulated progenitor LC proliferation, it delayed LC differentiation by targeting LC gene expression.

Apparently, the number of LC (CYP11A1^+^) cells, which serve as a biomarker for progenitor LCs [8], significantly increased at 1 and 2 mg/kg of patulin exposure. In contrast, no significant alteration was observed for HSD11B1^+^ (a biomarker of more mature LCs) [25], indicating that patulin primarily affects the proliferation of progenitor LCs. Notably, this study demonstrated an elevated proliferation rate of progenitor LCs following patulin exposure, as evidenced by the increased PCNA-labeling index [26] at 1 and 2 mg/kg. This finding implies that patulin promotes cell proliferation in the developing LC population. To elucidate the underlying mechanism of patulin-mediated proliferation, we examined the cell proliferation inhibition pathway (TP53 and CDKN1B) and cell proliferation stimulation pathways (AKT1, ERK1/2, and CREB). Patulin exposure resulted in significant reductions in the expressions of TP53 and CDKN1B, while the expression of CCND1 was significantly increased. Additionally, Patulin exposure activated signaling pathways involved in cell proliferation, including pAKT1, pERK1/2, and pCREB. These findings suggest that patulin stimulates cell cycle progression and proliferation through the activation of these signaling molecules.

In vitro experiments using an LC line (R2C) further validated the effects of patulin on cell proliferation. Patulin treatment resulted in increased EdU incorporation, indicating enhanced DNA synthesis and cell division. These results indicate that patulin is a stimulator of LC proliferation.

The increased proliferation may also be due to the increased level of LH due to negative feedback. LH is a stimulator of progenitor LCs via activating AKT1, ERK1/2, and CREB [27,28].

Another major action of patulin is the inhibition of progenitor LC differentiation. Indeed, gene expression analysis revealed that patulin downregulated *Lhcgr*, *Scarb1*, and *Cyp17a1* at 1 and 2 mg/kg. The downregulation of these genes further supports the disruption of steroidogenesis induced by patulin exposure. The in vitro R2C cell assay also showed that patulin inhibited testosterone secretion at 4 μM. Apparently, these effects of patulin on LC differentiation were within the testis, as judged by the decreased ratio of T/LH.

Furthermore, patulin exhibited a direct inhibitory effect on 11β-HSD2 activity, an enzyme responsible for the inactivation of active glucocorticoids [19]. Elevated glucocorticoids are able to inhibit LC development and function [21,29,30]. The inhibition of 11β-HSD in LCs could increase LC sensitivity to inhibition by active glucocorticoids [29]. This suggests that patulin may exert its adverse effects by increasing the availability of active glucocorticoids, which can disrupt LC function and steroidogenesis. However, caution should be raised for the inhibition of the 11β-HSD2 pathway, as the inhibitory potency of patulin on 11β-HSD2 was much weaker because the IC_50_ values were over 100 μM, which is much higher than the concentration of patulin needed to inhibit testosterone secretion (4 μM).

Previous studies have investigated the impact of patulin on the reproductive system of growing male rats, observing decreased sperm counts and abnormalities in sperm morphology, as well as histopathological changes in the epididymis and prostate tissue [22,24]. In this study, we studied the effects of patulin on the prepubertal development of LCs. We also found the novel mechanisms of patulin for stimulating the proliferation of progenitor LCs via activating AKT1 and CCND1 and inhibiting TP53/CDKN1B but inhibiting differentiation via downregulating the expression of Lhcgr, Scarb1, and Cyp17a1. We also found that patulin can directly inhibit 11β-HSD2, thereby contributing to the protection from the adverse effects of glucocorticoids, which inhibit LC differentiation [19]. The novelty of the data obtained in this study may lie in its focus on LC development and differentiation, as well as the identification of specific molecular pathways affected by patulin exposure. These findings could contribute to a better understanding of the toxic mechanisms of patulin on reproductive health, particularly in relation to LC function and the potential transgenerational effects on offspring.

**Figure 10:**
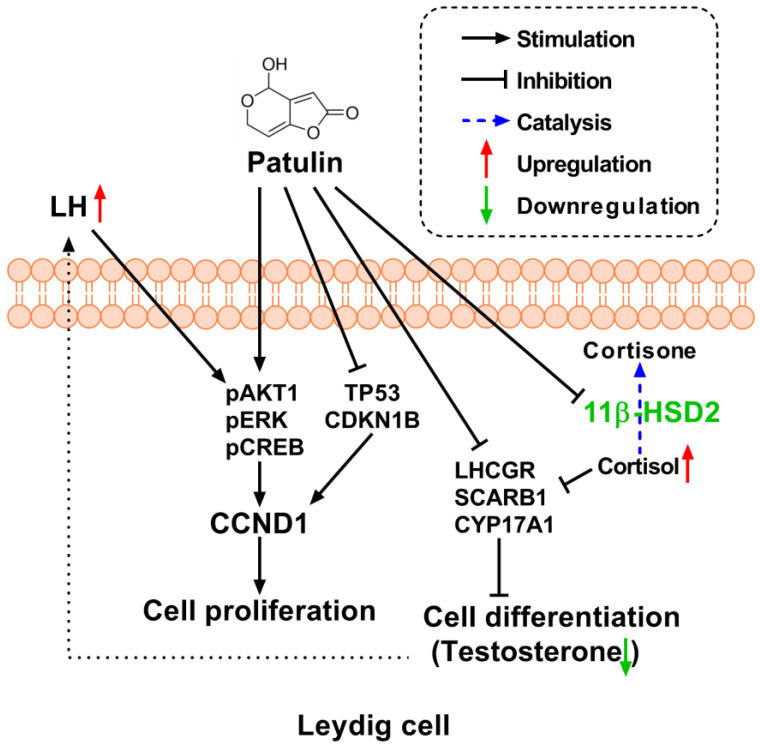
The mechanism of patulin action on main proteins in this study.

The findings of this study provide insights into the potential mechanisms by which patulin affects progenitor LC development in young male rats. Patulin reduces testosterone levels, promotes progenitor LC proliferation, and activates signaling pathways associated with cell cycle regulation and proliferation. These effects may contribute to the reproductive toxicity observed in patulin-exposed individuals (Figure 10).

## 4. Conclusions

Our study highlights the impacts of patulin on progenitor LC development, hormone synthesis, cell proliferation, and signaling pathways involved in steroidogenesis and proliferation. Understanding the mechanisms underlying the effects of patulin is crucial for assessing its reproductive toxicity and developing strategies to mitigate its harmful effects on reproductive health. Further research is needed to explore the long-term consequences of patulin exposure and the potential remedial measures to protect against its detrimental effects.

## 5. Materials and Methods

### 5.1. Materials and Animals

Patulin (CAS: 149-29-1; 98% purity; Figure 1A) was purchased from J&K Scientific (Beijing, China). Corticosterone, cortisone, corticosterone, and 11-deoxycorticosterone were obtained from Steraloids headquartered in Newport, RI, USA. Dimethyl sulfoxide (DMSO), phosphate-buffer saline (PBS, pH 7.2, 0.01 M), sucrose, acetonitrile, dithiothreitol, and nicotinamide adenine dinucleotide (NAD^+^) were purchased from Sigma (St. Louis, MO, USA), testosterone-d5 (T-d5) was from Zzbio (Shanghai, China), TRIzol from Invitrogen located in Carlsbad, CA, USA, Immulite2000 T progesterone kits from the China National Medical Supply Chain Service (Hangzhou, China), and rat LH and FSH ELISA kits from Jianglai (Shanghai, China). The ELISA kit for rat E2 was purchased from Westang (catalog no. F15321, Shanghai, China). Male Sprague Dawley rats (14 days of age in litters) were acquired from Shanghai Animal Center in China. The feed (normal chow, catalog no. mk031) for rats was purchased from China Sipeifu Biotechnology Co. (Beijing, China). The Institutional Animal Care and Use Committee of Wenzhou Medical University approved the animal experiments (wydw2023-0281), which were conducted following the Guide for the Care and Use of Laboratory Animals.

### 5.2. Animal Experiment

Each rat was assigned a unique numerical identifier from 1 to 24 and randomly allocated into four groups using a random number generator. Care was taken to ensure the random assignment of numbers within each group, minimizing potential correlations among the rats. Each group consisted of six rats, identified by their assigned numerical identifiers. The rats underwent a one-week adjustment period in a specific pathogen-free (SPF) room maintained at a temperature of 23 ± 2 °C, humidity ranging from 45 to 55%, and a 12 h light–dark cycle. From 21 days to 28 days postpartum, the rats were administered different doses of patulin (0, 0.5, 1, or 2 mg/kg/day) dissolved in water via oral gavage (Figure 1B). At the end of the experiment, the rats were euthanized using CO_2_ asphyxiation. Trunk blood was collected for serum analysis, and the testes were collected for further examination.

### 5.3. Measurement of Serum Hormones

Immulite2000 kits were used to measure serum testosterone and progesterone as previously described [31]. The minimum detection limit for testosterone and progesterone is 0.1 ng/mL. Serum LH and FSH levels were determined as described in [31], with a minimum detection limit of 0.1 IU/L for both LH and FSH. In the pre-coated wells, serum was added and incubated and then incubated with a secondary antibody for 1 h. After washing, substrates were added for 15 min and then the absorbance was read at 450 nm using a microplate reader.

### 5.4. Immunohistochemical Staining

The testes were fixed in Bouin’s solution for 24 h and then dehydrated and embedded as previously described [31]. CYP11A1 was used as an LC marker [31]. Because HSD11B1 only exists in immature LCs, not in progenitor LCs [25], this marker was also used for LC maturity. SOX9 was used as a Sertoli cell marker [32]. Immunohistochemical staining was carried out as previously described [31]. In brief, the slide was blocked, the antigen was unmasked, and CYP11A1, HSD11B1, or SOX9 antibodies were incubated, and then a secondary antibody was combined. Staining was shown, and Mayer hematoxylin was used as a counterstain. Non-immune rabbit IgG was used as the negative. The slide was scanned as a digital file with a Nano Zoomer XR scanner (Hamamatsu, Japan). Image-Pro Plus 7.0 software (Media Cybernetics, Silver Spring, MD, USA) was used for the analysis of CYP11A1^+^, HSD11B1^+^ LCs, and SOX9^+^ Sertoli cells, which were stereologically enumerated as previously described [33].

### 5.5. Double-Staining Immunofluorescence of PCNA and CYP11A1

Proliferating cell nuclear antigen (PCNA) can be used to label proliferating cells [26]. Double-staining immunofluorescence was carried out to determine the LC proliferation rate as previously described [17]. The slides were permeabilized with 0.1% Triton X-100. After primary antibody incubation, Alexa-conjugated secondary antibodies (Cell Signaling Technology, Boston, MA, USA) were followed to label CYP11A1 (green) and PCNA (red). The PCNA-labeling index was presented.

### 5.6. qPCR

Testicular RNA was extracted using a TRIzol kit. Subsequently, the cDNA was prepared using a reverse transcriptase kit (Vazyme, Nanjing, China). The transcripts were amplified using a qPCR kit (QIAGEN, Shanghai, China). LC mRNA *Lhcgr*, *Scarb1*, *Star*, *Cyp17a1*, *Cyp11a1*, *Hsd3b1*, *Hsd17b3*, *Srd5a1*, *Hsd11b1*, *Insl3*, and *Akr1c14* and Sertoli cell mRNA *Fshr*, *Dhh*, and *Sox9* as well as antioxidant *Sod1*, *Sod2*, *Gpx1*, and *Cat* mRNA were measured. Ribosomes S16 (Rps16) served as the internal control. Further information regarding the primers utilized can be found in Supplementary Table S1.

### 5.7. Western Blotting

Protein was extracted from the testes using RIPA buffer (Cat# P0013B, Beyotime, Shanghai, China) as previously described [31]. The protein concentration was determined using a BCA protein assay kit. Denaturation and electrophoresis were performed, followed by transferring to a membrane and sequential incubation with the primary antibody, secondary antibody, and chemiluminescence substrate. The following antigens were identified: LHCGR, SCARB1, CYP17A1, TP53, CDKN1B, CCND1, AKT1, phospho-AKT1 (pAKT1), ERK1/2, phospho-ERK1/2 (pERK), CREB, phospho-CREB (pCREB), and ACTB. The intensity of the protein was measured and quantified using Image lab software (Version 3.0) (Bio-Rad, Hercules, CA, USA) and normalized to the housekeeping protein ACTB. The antibody information is provided in Supplementary Table S2.

### 5.8. The Measurement of Malondialdehyde (MDA)

The testicular MDA content was determined as previously described [34]. In brief, a homogenate of the testis was prepared using an MDA kit (Solibao Biotechnology, Shanghai, China) following the manufacturer’s instructions. The amount of MDA (mmol/g testis) was calculated with the following Formula: 5 × (12.9 × (ΔA532 − ΔA600) − 2.58 × Δ450)/0.1.

### 5.9. Rat LC Line R2C Culture

The R2C cell, derived from rat LC carcinoma and obtained from the ATCC in the Manassas, VA, USA, was cultured according to standard protocols. Specifically, the R2C cells were cultured in RPMI-1640 medium supplemented with 10% FCS (Invitrogen, Carlsbad, CA, USA) in a 5% CO_2_ incubator at 37 °C. To conduct experiments, exponentially growing cells with an 80% confluence were exposed to varying concentrations of patulin (0, 1, 2, or 4 μM) for 24 h. The culture medium was then collected for the measurement of the testosterone concentration.

### 5.10. Flow Cytometric Analysis of R2C Cell Cycle

Propidium iodide (PI) is a fluorescent DNA stain that binds to DNA and emits fluorescence proportional to the DNA content. After treating LCs with patulin for 24 h, the cells were collected and fixed with ethanol. Subsequently, a buffer containing PI was incubated at 37 °C for 30 min, allowing the formation of a complex between the DNA in the cell nucleus and PI. The fluorescence intensity of PI was then detected using flow cytometers (BD, Franklin Lakes, NJ, USA), which enabled the determination of the DNA content in the cells. By analyzing changes in cell DNA content, different stages of the cell cycle can be identified.

### 5.11. EdU Incorporation into R2C Cells

To determine the effect of patulin on LC proliferation, EdU incorporation into R2C cells was utilized with the EdU Alexa Fluor Kit (Life Technologies, Carlsbad, CA, USA). The R2C cells were treated with 0–4 μM patulin for 24 h as detailed above. EdU (diluted 1:1000, *v/v*) was incubated for an additional 24 h, followed by fixation in 4% paraformaldehyde for 30 min and reaction for the display labeled nucleus (green). LCs were visualized via the immunofluorescence of CYP11A1 (red) and 4’,6-diamidino-2-phenylindole (DAPI) (Sigma-Aldrich, St. Louis, MO, USA). Image acquisition was performed using a Leica automatic stage DM5500B microscope (Leica, Wetzlar, Germany), and the EdU-positive cells were counted using Image Pro Plus 7.0 software.

### 5.12. Measurement of HSD11B2 Activity and Molecular Docking

Previous studies indicated that glucocorticoid corticosterone can inhibit LC development and function [29,35], and 11β-hydroxysteroid dehydrogenase 2 (HSD11B2) is present in LCs [19], converting the biologically active glucocorticoid into inert 11-keto glucocorticoids to protect LCs [19]. To explore whether patulin can directly inhibit rat and human HSD11B2 activity, HSD11B2 in rat testicular microsomes and human placental microsomes (Human full-term placentas obtained from the Second Affiliated Hospital of Wenzhou Medical University under protocol (2022-K-81-01) approved by the Clinical Research Committee of the Second Affiliated Hospital, Wenzhou Medical University.) were used as sources. Rat testicular microsomes were prepared as previously described [36]. In brief, a piece of testis was homogenized in cold 0.01 M PBS containing 0.25 M sucrose and centrifuged at 700× *g* for 30 min. The supernatant was transferred to a new tube and then centrifuged at 10,000× *g* for 30 min, followed by 105,000× *g* twice for 60 min to obtain a microsomal pellet. The protein concentration was measured using the Bio-Rad Protein Assay Kit (Cat No. 500-0006, Bio-Rad, Hercules, CA, USA) according to the manufacturer’s protocol. The measurement of HSD11B2 was performed as previously described [37]. In brief, each 100 μL of enzyme reaction buffer (PBS; 0.01 M; pH 7.2) contained 5 μg of human or rat microsomes, 25 nM substrate (cortisol for human and corticosterone for rat HSD11B2), 0.2 mM NAD^+^, 0.1 mM dithiothreitol, and a range of concentrations of patulin and was incubated for 30 min. At the end of the reaction, it was terminated by adding 200 μL of acetonitrile and 10 μM testosterone-d5 (as an internal control), and steroids were extracted as an organic supernatant, which (6 μL) was injected into an HPLC-MS/MS system (Waters, Milford, MA, USA), which contained Acquity Ultra Performance Liquid Chromatography equipment and an Acquity BEH C18 column (2.1 mm; 50 mm; 1.7 μm) and a 0.2 mm stainless steel frit filter, for the measurement of the cortisone or 11-dehydrocorticosterone product. The mobile phase was solvents A (0.1% formic acid in water) and B (acetonitrile). The gradient program for the mobile phase was set as 5–5% solution B (0–0.5 min), 5–95% solution B (0.5–1.0 min), 95–95% solution B (1.0–2.0 min), and 95–5% solution B (2.0–2.1 min). For cortisol and IS, the multiple reaction monitoring mode transitions were *m/z* 358.3340.3 and *m/z* 237.2194.3, respectively. The conversion of substrate to product was calculated. The residual activity under the control (DMSO) was set as 100%. The dose–response (three parameters) relationship was calculated via the nonlinear regression of the inhibitor vs. response for three parameters in GraphPad (version 8, San Diego, CA, USA) for the half-maximum inhibitory concentration (*IC*_50_) value of patulin to inhibit human and rat HSD11B2 using the following equation:

(1) $$Y=Bottom+\frac{Top-Bottom}{1+\frac{x}{IC_{50}}}$$

where *Y* is the residual activity, *Bottom* is zero, and *X* is the concentration of patulin. To explore the underlying mechanism of inhibiting HSD11B2, homology model structures of human and rat HSD11B2 enzymes were obtained from the Swiss-Model database, which is based on the crystal structure of human estrogenic 17β-hydroxysteroid dehydrogenase 1 (https://swissmodel.expasy.org/repository/uniprot/P80365, accessed on 4 September 2022, 1bhs.1.B [38]) for human HSD11B2, and on the crystal structure of carveol dehydrogenase from Mycobacterium avium (https://swissmodel.expasy.org/repository/uniprot /P50233, accessed on 8 August 2022, 3uve.1.A [39]) for rat HSD11B2. Autodock v4 software was used to perform the molecular docking analysis of patulin with these two enzymes, and the best conformation of patulin’s structure with the lowest binding energy (ΔG) was picked and displayed using PyMOL software (Version 2.6.0a0) and LigPlot as previously described [40]. Ki (inhibitory constant) is a measure of the binding affinity between a ligand and its target protein. It represents the concentration of a ligand required to achieve 50% inhibition of the target protein’s activity. The Ki value was estimated using Autodock4 software (Version 4.2.6) after a complex computational method.

### 5.13. Statistical Analysis

The means ± standard error of the means (SEMs) were used to present the data. Statistical significance was determined as *p* < 0.05. To compare with the control, a one-way ANOVA was conducted, followed by post hoc Dunnett’s multiple comparisons using GraphPad (version 8, GraphPad Software Inc., San Diego, CA, USA).

## Figures and Tables

**Table 1:** Toxicological observations after patulin administration.

Parameter	Dose of Patulin (mg/kg/day)			
	0	0.5	1	2
Body weight (g) Before treatment	88.5 ± 3.24	91.0 ± 3.72	86.8 ± 2.73	91.5 ± 4.10
Body weight (g) After treatment	135.8 ± 5.86	137.2 ± 6.54	136.3 ± 3.63	142.7 ± 6.79
Change in body weight (g)	44.0 ± 5.01	39.0 ± 5.59	45.3 ± 0.71	44.5 ± 5.60
Testis weight (g)	0.54 ± 0.03	0.56 ± 0.02	0.55 ± 0.03	0.60 ± 0.02
Gonadosomatic index (GSI, %)	0.40 ± 0.03	0.41 ± 0.01	0.40 ± 0.02	0.43 ± 0.02

Mean ± SEM; n = 6. No statistically significant difference between patulin and the vehicle control was observed.

## Data Availability

Data is contained within the article or Supplementary Material.

## Supplementary Materials

The following supporting information can be downloaded at: https://www.mdpi.com/article/10.3390/toxins15090581/s1: Table S1: Primer information for quantitative polymerase chain reaction, Table S2: Antibodies information for Western blot, immunohistochemistry, and immunofluorescence, Figure S1: Effects of patulin on mRNA expression in rat testis, and Figure S2: Effects of patulin on antioxidant mRNA expression in rat testis and MDA levels.
