# Supplementary material for: Patulin Stimulates Progenitor Leydig Cell Proliferation but Delays Its Differentiation in Male Rats during Prepuberty
Source: Toxins (Basel). 2023 Sep 20;15(9):581. doi: 10.3390/toxins15090581 (PMC10538017; doi:10.3390/toxins15090581)
Supplement: Supplementary file 1 [file toxins-15-00581-s001.zip › toxins-2581822-supplementary.pdf]

toxins

Supplementary Materials: Patulin Stimulates Progenitor Leydig Cell Proliferation but Delays Its Differentiation in Male Rats during Prepuberty

Huitao Li [1,2,3,†], Ming Su [1,†], Hang Lin [1], Jingjing Li [1], Shaowei Wang [1], Lei Ye [1], Xingwang Li [1] and Renshan Ge [1,2,3,*]

1 Department of Anesthesiology and Perioperative Medicine, The Second Affiliated Hospital and Yuying Children's Hospital, Wenzhou Medical University, Wenzhou 325027, China; lihuitao1991@163.com (H.L.); suming2000@163.com (M.S.); lh1014794144@163.com (H.L.); lbling68@163.com (J.L.); smilewei9898@163.com (S.W.); leiye3567@163.com (L.Y.); li-xingwang@163.com (X.L.)
2 Key Laboratory of Pediatric Anesthesiology, Ministry of Education, Wenzhou Medical University, Wenzhou 325027, China
3 Key Laboratory of Environment and Male Reproductive Medicine of Wenzhou, and Key Laboratory of Structural Malformations in Children of Zhejiang Province, The Second Affiliated Hospital and Yuying Children's Hospital, Wenzhou Medical University, Wenzhou 325027, China
* Correspondence: renshan_ge@wmu.edu; Tel.: +86-577-88002198
† These authors contributed equally to this work.

Supplementary Table S1. Primer information for quantitative polymerase chain reaction.

Primer Symbol	Gene Name	Primer Direction	Sequences (5′to 3′)	PCR (bp)	Accession
Lhcgr	Luteinizing hormone receptor	Forward	CTGCGCTGTCCTGGCC	102	NM_012978
		Reverse	CGACCTCATTAAGTCCCCTGAA		
Scarb1	Scavenger receptor class B, member 1	Forward	ATGGTACTGCCGGGCAGAT	117	NM_031541
		Reverse	CGAACACCCTTGATTCCTGGTA		
Star	Steroidogenic acute regulatory protein	Forward	CCCAAATGTCAAGGAAATCA	187	NM_031558
		Reverse	AGGCATCTCCCCAAAGTG		
Cyp11a1	Cholesterol side chain cleavage enzyme	Forward	AAGTATCCGTGATGTGGG	127	NM_017286
		Reverse	TCATACAGTGTCGCCTTTTCT		
Hsd3b1	3β-Hydroxysteroid dehydrogenase 1	Forward	CCCTGCTCTACTGGCTTGC	189	NM_001007719
		Reverse	TCTGCTTGGCTTCCTCCC		
Cyp17a1	P450 17α-hydroxylase/ 17,20-lyase	Forward	TGGCTTTCCTGGTGCACAATC	90	NM_012753
		Reverse	TGAAAGTTGGTGTTCGGCTGAAG		
Hsd11b1	Hydroxysteroid 11β-dehydrogenase 1	Forward	GAAGAAGCATGGAGGTCAAC	133	NM_017080
		Reverse	GCAATCAGAGGTTGGGTCAT		
Hsd17b3	17β-Hydroxysteroid dehydrogenase 3	Forward	TGAAAGTTGGTGTTCGGCTGAAG	202	NM_054007
		Reverse	CCACAAGCCAATACAAACTAACT		
Insl3	Insulin-like 3	Forward	GTGGCTGGAGCAACGACA	102	NM_053680
		Reverse	AGAAGCCTGGTGAGGAAGC		
Srd5a1	5α-Reductase 1	Forward	TCACCAGAGCGAAGCAGC	92	NM_017070
		Reverse	CTAAAGCACAAATGGAATCAGTAT		
Akr1c14	3α-Hydroxysteroid dehydrogenase	Forward	GCAGCGTGGGGTTGTG	172	NM_138547
		Reverse	TGGATGATTGGGATGGTCA		
Sox9	SRY box 9	Forward	TGCTGAACGAGAGCGAGAAG	160	NM_080403
		Reverse	ATGTGAGTCTGTTCGGTGGC		
Dhh	Desert hedgehog	Forward	AACCCCGACATAATCTTCA	150	NM_053367
		Reverse	CTCGTCCCAACCTTCAGT		
Fshr	Follicle-stimulating hormone receptor	Forward	CCACAAGCCAATACAAACTAACT	327	NM_199237
		Reverse	CAAAAGTCCAGCCCAATACC		

Sod1	Superoxide dismutase 1	Forward	GCCGTGTGCGTGCTGAAGG	99	NM_011434
		Reverse	TGTAATCTGTCCTGACACCACAACTG		
Sod2	Superoxide dismutase 2	Forward	TCCCTGACCTGCCTTACGACTATG	130	NM_013671
		Reverse	TCGTGGTACTTCTCCTCGGTGAC		
Gpx1	Glutathione peroxidase 1	Forward	TGCAATCAGTTCGGACATCAGGAG	129	NM_001329528
		Reverse	CTCACCATTCACCTCGCACTTCTC		
Cat	Catalase	Forward	AGCGGATTCCTGAGAGAGTGGTAC	147	NM_012520
		Reverse	CTGTGGAGAATCGGACGGCAATAG		
Rps16	Ribosomal protein S16	Forward	AAGTCTTCGGACGCAAGAAA	148	NM_001169146
		Reverse	TTGCCCAGAAGCAGAACAG		

Table S2. Antibodies information for Western blot, immunohistochemistry and immunofluorescence.

Antibody	Species	Vendor (City, State,)	Dilution		
			WB	IHC	IF
ACTB	Rabbit	Bioworld Technology (Minnesota, CA)	1:10000	ND	ND
CYP11A1	Rabbit	Cell Signaling Technology (Danvers, MA)	1:1000	1:400	ND
HSD11B1	Rabbit	Abcam (San Francisco, CA)	1:1000	1:200	ND
SOX9	Rabbit	Cell Signaling Technology (Danvers, MA)	ND	1:200	ND
PCNA	Mouse	Abcam (San Francisco, CA)	ND	ND	1:200
LHCGR	Rabbit	Abcam (San Francisco, CA)	1:1000	ND	ND
SCARB1	Rabbit	Abclonal (Wuhan, CN)	1:1000	ND	ND
CYP17A1	Rabbit	Abcam (San Francisco, CA)	1:10000	ND	ND
pAKT1	Rabbit	Abcam (San Francisco, CA)	1:5000	ND	ND
AKT1	Rabbit	Abcam (San Francisco, CA)	1:5000	ND	ND
P53	Rabbit	Cell Signaling Technology (Danvers, MA)	1:1000	ND	ND
CREB	Rabbit	Abcam (San Francisco, CA)	1:1000	ND	ND
pCREB	Rabbit	Abcam (San Francisco, CA)	1:5000	ND	ND
CCND1	Rabbit	Proteintech Group,Inc. (Chicago，CA)	1:10000	ND	ND
P27	Rabbit	Abclonal (Wuhan, CN)	1:1000	ND	ND
$ERK_{1/2}$	Mouse	Abcam (San Francisco, CA)	1:10000	ND	ND
$pERK_{1/2}$	Mouse	Abcam (San Francisco, CA)	1:1000	ND	ND

ND = Not detected; WB = Western blot; HS = Histochemical staining; IHC = Immunohistochemistry; IF = Immunofluorescence.

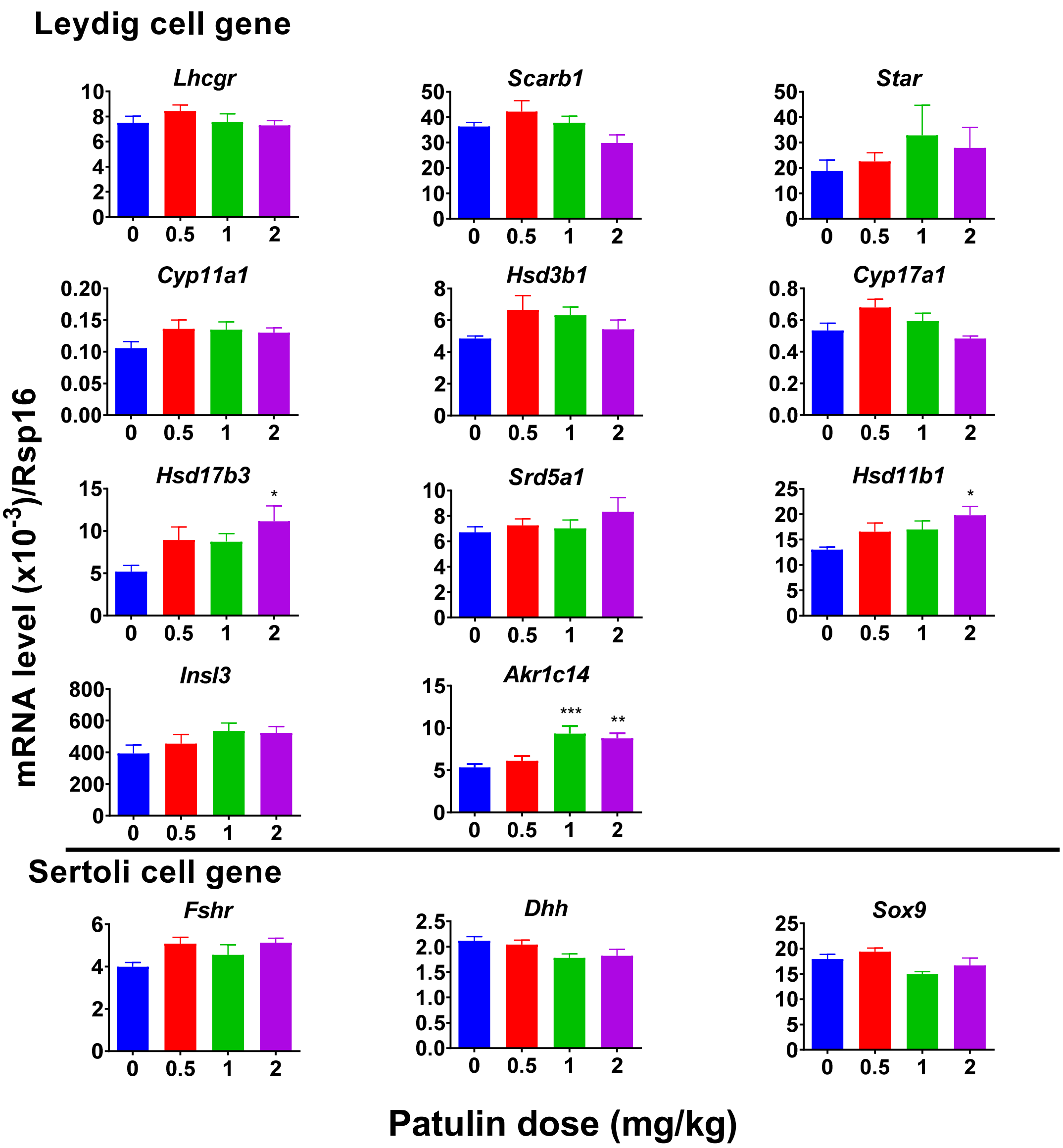

Figure S1. Effects of patulin on mRNA expression of rat testis. Mean ± SEM, n = 6. * $p < 0.05$, ** $p < 0.01$, *** $p < 0.001$ show significant differences from the control group (patulin 0 mg/kg/day).

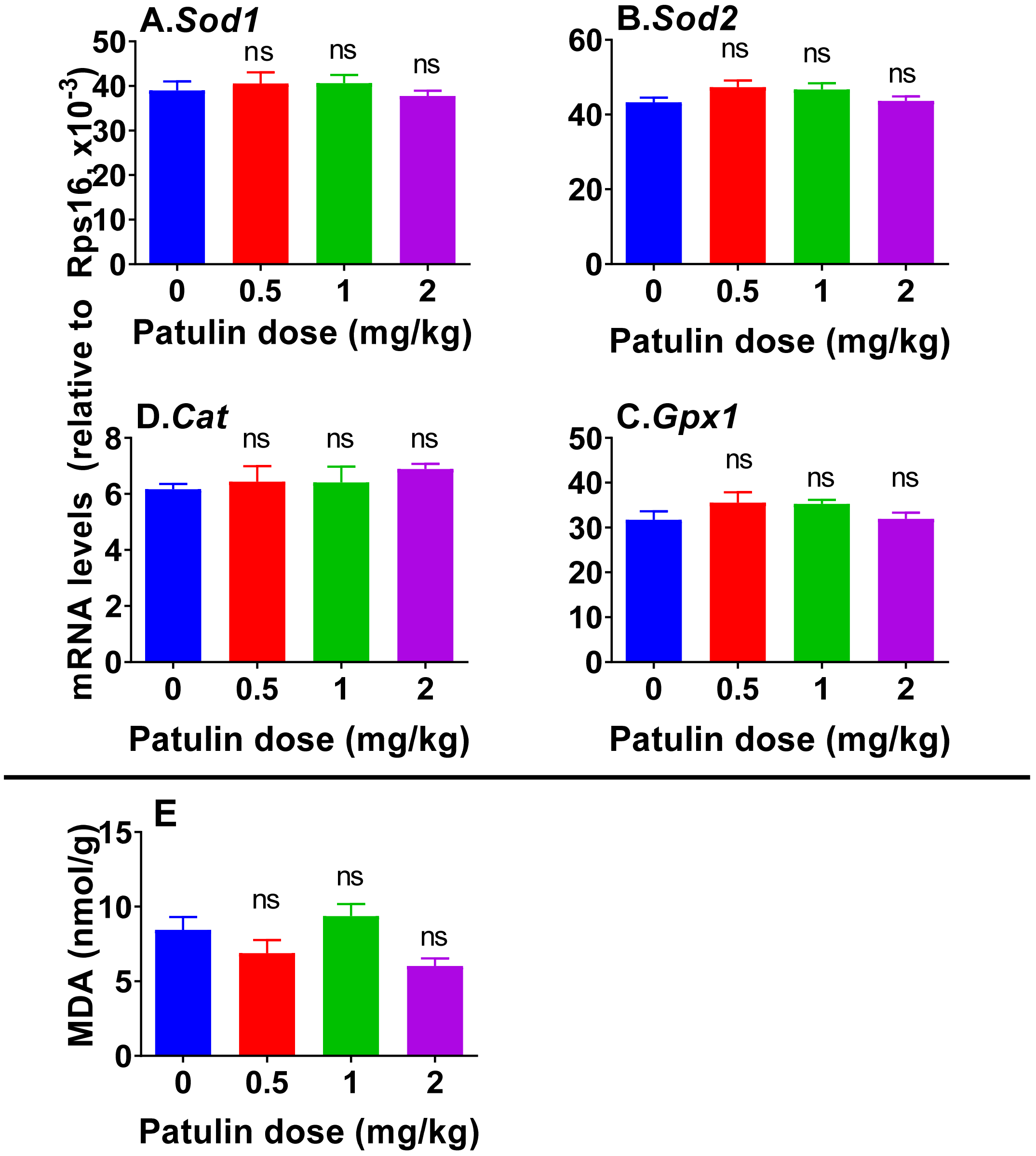

Figure S2. Effects of patulin on antioxidant mRNA expression of rat testis and MDA levels. Mean ± SEM, n = 6. ^{ns} $p > 0.05$ show significant differences from the control group (patulin 0 mg/kg/day).
